# Supplementary material for: Towards unified AI-driven fracture mechanics: the extended deep energy method (XDEM)
Source: Nat Commun. 2026 Aug 15;17:8492. doi: 10.1038/s41467-026-76748-1 (PMC13484471; doi:10.1038/s41467-026-76748-1)
Supplement: Supplementary file 3 [file 41467_2026_76748_MOESM3_ESM.zip › pic/Results/Crack_propagation/Straight/3D_single_crack.pdf]

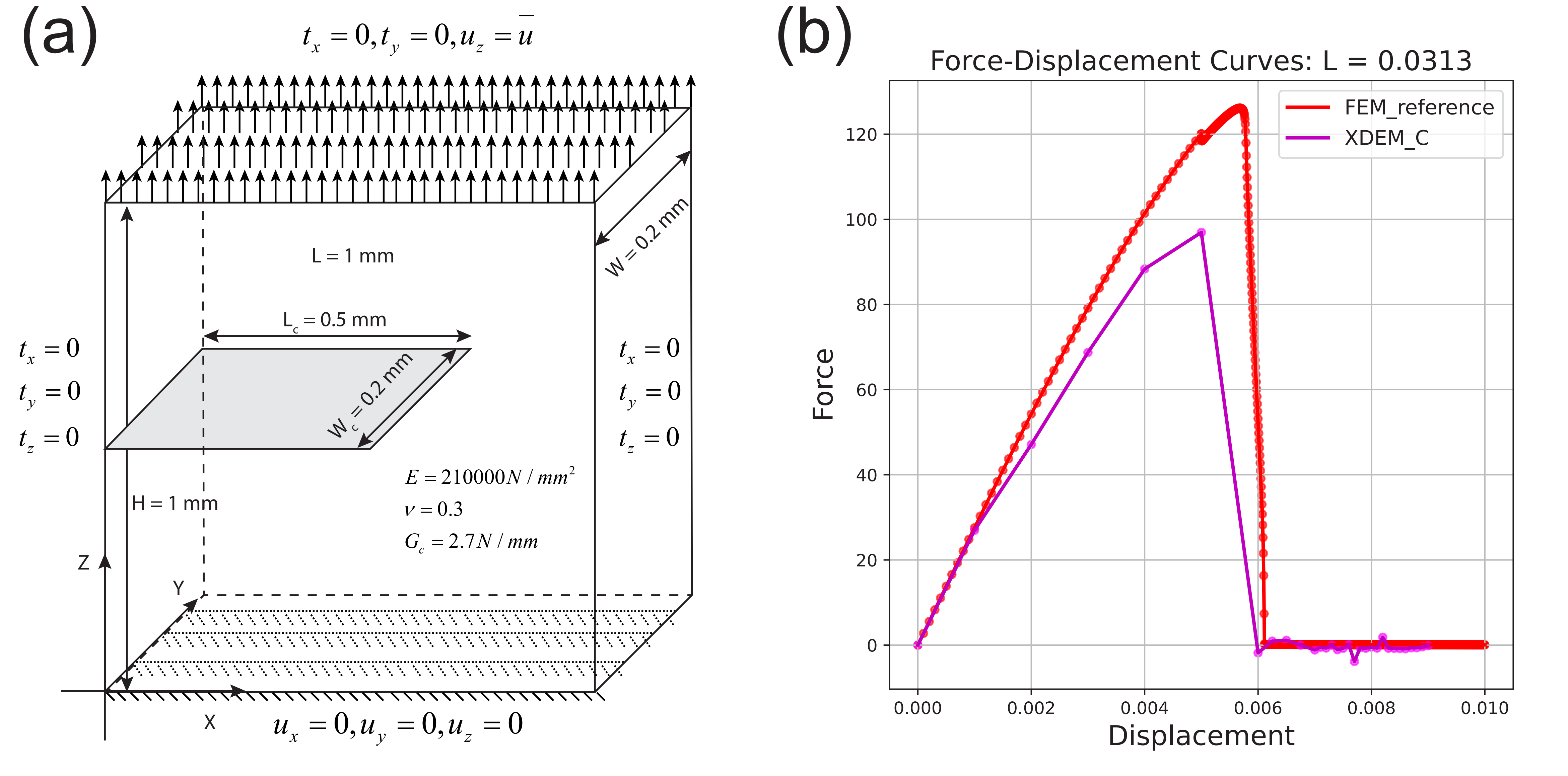
(a)
$t_x = 0, t_y = 0, u_z = \bar{u}$
L = 1 mm
W = 0.2 mm
L_c = 0.5 mm
W_c = 0.2 mm
$t_x = 0$
$t_y = 0$
$t_z = 0$
$E = 210000 N/mm^2$
$\nu = 0.3$
$G_c = 2.7 N/mm$
H = 1 mm
Z
Y
X
$u_x = 0, u_y = 0, u_z = 0$
(b)
Force-Displacement Curves: L = 0.0313
FEM_reference
XDEM_C
Force
Displacement
120
100
80
60
40
20
0
0.000
0.002
0.004
0.006
0.008
0.010
